# An isomiR expression panel based novel breast cancer classification approach using improved mutual information

**DOI:** 10.1186/s12920-018-0434-y

**Published:** 2018-12-31

**Authors:** Chaowang Lan, Hui Peng, Eileen M. McGowan, Gyorgy Hutvagner, Jinyan Li

**Affiliations:** 1 Advanced Analytics Institute, Faculty of Engineering and IT, University of Technology Sydney, PO Box 123, Broadway, NSW, 2007 Australia; 2 School of Life Sciences, University of Technology Sydney, PO Box 123, Broadway, NSW, 2007 Australia; 3 School of Biomedical Engineering, Faculty of Engineering and IT, University of Technology Sydney, PO Box 123, Broadway, NSW, 2007 Australia

**Keywords:** IsomiR, Improved mutual information, Breast cancer subtype

## Abstract

**Background:**

Gene expression-based profiling has been used to identify biomarkers for different breast cancer subtypes. However, this technique has many limitations. IsomiRs are isoforms of miRNAs that have critical roles in many biological processes and have been successfully used to distinguish various cancer types. Biomarker isomiRs for identifying different breast cancer subtypes has not been investigated. For the first time, we aim to show that isomiRs are better performing biomarkers and use them to explain molecular differences between breast cancer subtypes.

**Results:**

In this study, a novel method is proposed to identify specific isomiRs that faithfully classify breast cancer subtypes. First, as a null hypothesis method we removed the lowly expressed isomiRs from small sequencing data generated from diverse breast cancers types. Second, we developed an improved mutual information-based feature selection method to calculate the weight of each isomiR expression. The weight of isomiR measures the importance of a given isomiR in classifying breast cancer subtypes. The improved mutual information enables to apply the dataset in which the feature is continuous data and label is discrete data; whereby, the traditional mutual information cannot be applied in this dataset. Finally, the support vector machine (SVM) classifier is applied to find isomiR biomarkers for subtyping.

**Conclusions:**

Here we demonstrate that isomiRs can be used as biomarkers in the identification of different breast cancer subtypes, and in addition, they may provide new insights into the diverse molecular mechanisms of breast cancers. We have also shown that the classification of different subtypes of breast cancer based on isomiRs expression is more effective than using published gene expression profiling. The proposed method provides a better performance outcome than Fisher method and Hellinger method for discovering biomarkers to distinguish different breast cancer subtypes. This novel technique could be directly applied to identify biomarkers in other diseases.

## Background

MicroRNAs (miRNAs) are short RNA molecules and play vital regulatory roles in a variety of biological processes [1]. Mature miRNAs are generated from longer transcripts via several sequential processing steps [2]. First the primary miRNA transcripts (pri-miRNA) are cleaved by the Microprocessor complex that contains Drosha, an RNase III enzyme [3]. The cleaved precursor miRNAs (pre-miRNA) are further processed by another RNase III enzyme, Dicer, to produce small miRNA duplexes [4]. Alterations in miRNA maturation, such as the alternative and imprecise cleavage of Drosha and Dicer, or the turnover of miRNAs could result in miRNAs that are heterogeneous in length and/or sequence [5, 6]. These variants are called isomiRs (isoforms of miRNA) and can be divided into three main categories: 3^′^ isomiR (trimmed or addition of one or more nucleotides at the 3^′^ position), 5^′^ isomiR (trimmed or addition of one or more nucleotides at the 5^′^ position), and polymorphic isomiR (some nucleotides within the sequence are different from the wild type mature miRNA sequence) [6].

It could be envisioned that the increased expression of miRNA variants, or individual isomiRs, lead to the loss or weakening of the function of the corresponding wild type mature miRNA or result in the regulation of a different transcriptome. Recent studies suggest that isomiRs probably play vital roles in a variety of cancers, tissues, and cell types [7]. For example, Juzenas and colleagues claimed that isomiRs are differentially expressed in different human blood cell types [8]. Telonis and colleagues showed that specific isomiRs could be superior cancer biomarkers compared to mature miRNAs when they used isomiRs to classify 32 different cancers [9]. Specifically, Telonis and colleagues demonstrated that miRNA-based analysis was unable to differentiate two specific subtypes of breast cancer while, in comparison, isomiRs were able to make clear distinctions between the two subtypes [10]. These findings suggest that isomiRs may play critical roles in differentiating subtypes of breast cancer and, furthermore, may provide novel insights into understanding the molecular mechanisms leading to the development of breast cancers.

Breast cancer is the most common cancer and the second leading cause of cancer-related deaths among women worldwide [11]. Routine clinical evaluation and diagnosis of breast cancer is categorised into three major distinct molecular subtypes based on their hormone receptor status: estrogen receptor (ER *α*) and progesterone receptor (PR) positive, Herceptin 2 positive (HER2+), and triple negative (ER/PR/HER2 negative) [12–14]. However, the link between molecular mechanisms and disease prognosis defining the breast cancer subtypes is unclear [15]. Understanding the mechanisms of breast cancer subtyping is clinically useful with respect to prognosis, prediction, and informed therapeutic choices [16]. Within the major breast cancer subtypes, gene expression profiling has been used to further classify these molecular subtypes with the potential to design more specific targeted therapies [17]. In addition, gene expression profiling has been found to be more predictive of treatment response. For example, in a study by Finn and colleagues they showed reclassification of breast cancer subtypes using an unbiased gene expression profiling technique predicted a better treatment outcome compared to the conventional breast cancer subtyping (ER/HER2 status) [18]. In this study, a subset of three genes expressed in breast cancer were more likely to predict responsiveness to dasatinib, a small molecule specific kinase inhibitor. Dasatinib has been used in clinical trials for hard to treat metastatic breast cancer [19]. However, most breast cancer clinical trial studies using dasatinib are inconclusive and potentially these studies would benefit from gene profiling to understand the lack of responsiveness.

Complex genetic diseases, such as breast cancer, inherently pose the problem to be characterised by a few biomarkers that faithfully characterise the subtypes of the disease. MiRNAs and isomiRs provide a potentially better alternative for classifying complex diseases compared to mRNA based biomarkering since they are regulatory “hubs” of gene expression. Therefore, the changes in their expression could influence multiple downstream mRNAs and therefore diverse biological pathways.

In this paper, we present a novel method that applies isomiR expression profiles for improved classification of breast cancer types using small RNA sequencing data available in the TCGA database. Firstly, since the TCGA dataset has many lowly expressed isomiRs that have significant negative influence on the identification of biomarkers, these lowly expressed isomiRs should be removed. The traditional method for removing the lowly expressed isomiRs is by selecting a ‘hard’ threshold [8, 9]. If the expression levels of an isomiR is lower than this ‘hard’ threshold, this isomiR is viewed as lowly expressed and should be removed. However, this ‘hard’ threshold may lead to a loss of information [20]. In order to tackle this disadvantage, a ‘soft’ method based on a null hypothesis method was applied, and this method was designed to remove these lowly expressed isomiRs. Secondly, we utilized an improved mutual information method to calculate the weight of each isomiR, which measured the significance of the isomiR to classify different subtypes of breast cancer. The higher the weight of the isomiR, the more suitable the isomiR for classifying different subtypes of breast cancer. The traditional mutual information can only be used if both the feature and the label are continuous or discrete data. This improved mutual information can be applied to features if it is continuous data and the label is discrete data. Finally, a few isomiRs, which have high weights, were able to classify different breast cancer subtypes. In order to identify these key isomiRs, the SVM classification method was used.

Although there are many methods that have been designed for biomarker discovery, they can be divided into two major categories. The first category selects a set of biomarkers that can classify the data [21], such as support vector machine (SVM) [22], mutual information [23], and swarm optimizer [24]. These methods do not calculate the weight of each biomarker and therefore, the importance of the biomarker in each breast cancer subtype classification is not known. The weight of the biomarker may reflect its regulatory importance in the molecular mechanism of the disease; therefore, it may be worth studying the potential role of gene regulation of highly weighed biomarkers. Another category of methods view the gene or isomiR as the feature and calculates the weight of each feature. The weight of the feature measures the importance of the feature in the classification. The top N features viewed as biomarkers. Information gain, t-test, and fold change methods are widely applied to identify biomarkers [25]. However, t-tests and fold change methods are not suitable for identifying biomarkers from the data that has more than three categories. Although the information gain can be applied to find biomarkers from multiple categories, this method is very time consuming. Other methods, such as Fisher [26] and correlation coefficient method [27], can calculate the weight of each feature for data that comprises of more than two categories and is less time consuming than information gain. However, these methods also have their limitations. The Fisher method is based on the mean and standard deviation of the dataset and therefore, small data sets, confounded by outliers will negatively influence the results. If weights of the feature are calculated by the correlation coefficient method, it challenges the rank features based on their weights [28]. Together, all these methods used for identifying biomarkers have their limitations. Therefore, a novel method is needed to identify unique, more discrete and effective biomarkers.

## Method

**Fig. 1:**
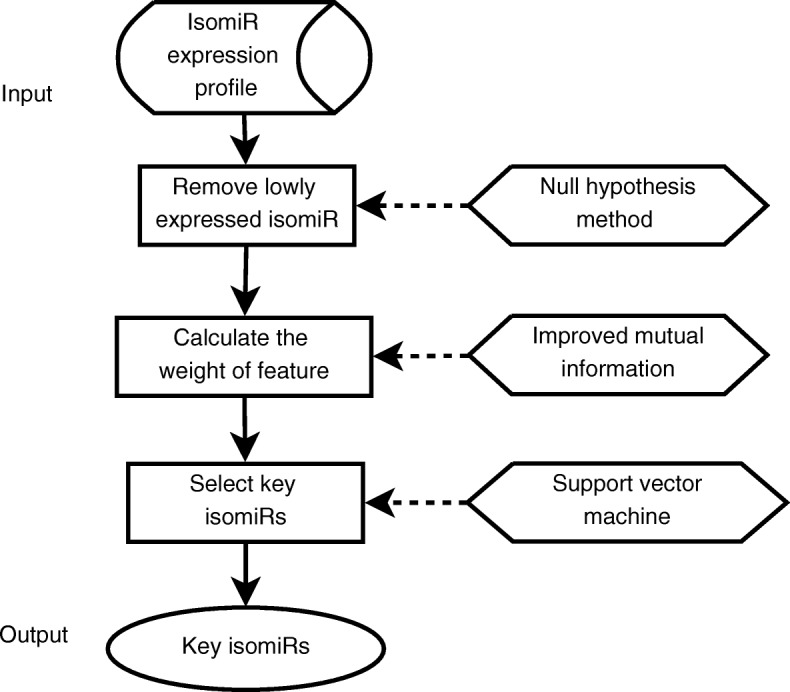
IsomiR biomarker subtyping methodology. The framework of the novel methodology designed for breast cancer biomarker subtyping is composed of three discrete steps from isomiR expression profiling to identification of key isomiRs used as novel biomarkers

Our method for identifying isomiR biomarkers in different subtypes of breast cancer is composed of three steps. Firstly, it computes the expression level of isomiRs in each breast cancer sample and removes the lowly expressed isomiRs. Secondly, it utilizes improved mutual information to calculate the weight of each isomiR. Finally, the third step selects the critical isomiRs for breast cancer subtype classification, for which the SVM classification method is applied. These key isomiRs are viewed as breast cancer subtype biomarkers. Figure 1 shows the framework of our methodology.

### Data source and definitions

The expression profiles of isomiRs in breast cancer patients can be downloaded from TCGA GDC data portal website (https://portal.gdc.cancer.gov). However, the website does not provide the name of each isomiR. The nomenclature used in this study for discrete isomiR was derived from its mature miRNA: the name of the isomiR comprises of the name of the corresponding wild type (referenced) miRNA followed by a variant symbol, e.g hsa-miR-21-5p | 3^′^t-2. The sign | separates the isomiR name into miRNA name and variant symbol. The variant symbol is divided into two parts by the sign (−). The first part indicates the variant type of the isomiR. 3^′^t (5^′^a) implies that this isomiR is 3^′^ trimming (5^′^ additional) isomiR. The second part denotes the number of the nucleotide that is trimmed or added. In addition, the number of reads are not suitable for analyze. Thus, we calculated the RPM (reads per million mapped reads) of each isomiR. The clinical information of the breast cancer patients was obtained from the website (https://www.nature.com/articles/nature11412#supplementary-information). Since the TCGA website does not provide the expression levels of polymorphic isomiRs, this kind of isomiR was not taken into consideration in this paper. Although the clinical information contained 824 breast cancer patients, only 698 patients had valid clinical information. In this paper, we applied these 698 patients’ isomiR expression levels to identify biomarkers that classify breast cancer subtypes.

The traditional clinical classification method sorts breast cancer into three different subtypes based on the hormone receptor status. However, some breast cancer patients proved to be positive in both ER *α*/PR+ and HER2+ receptor status. These breast cancer patients were identified as ER *α*/PR+ or HER2+ breast cancer subtypes. However, it was not suitable to classify these breast cancer patients as ER *α*/PR+ or HER2+ breast cancer subtype patients. Therefore, these patients were reclassified as a fourth breast cancer subtype. Together, the breast cancer patients were classified into four subtypes and the number of patients in each subtype of breast cancer are shown in Table 1.

**Table 1:** Breast cancer subtype reclassification for isomiR identification

Subtype name	ER *α*+HER2-	ER *α*-HER2+	ER *α*+HER2+	Triple negative
Number of patient	472	31	76	119

### Removal of lowly expressed isomiR

**Fig. 2:**
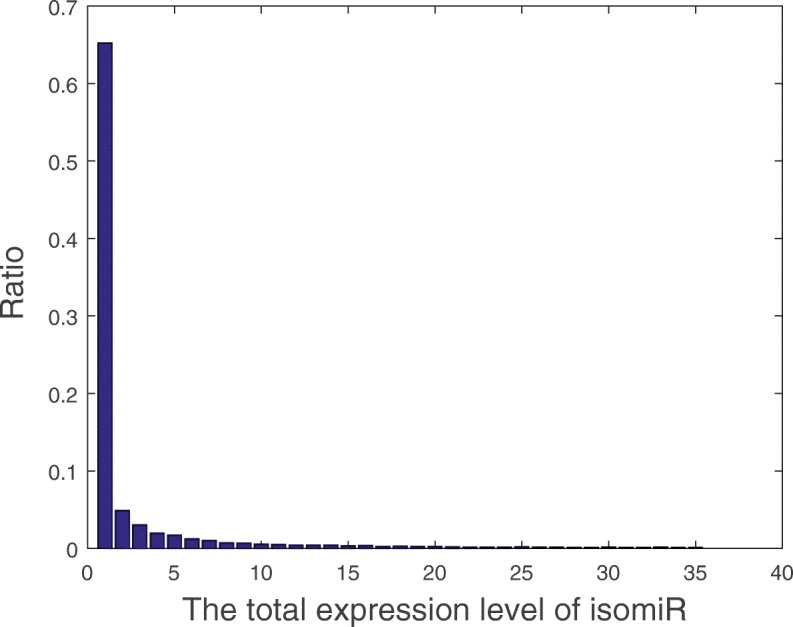
The distribution of total expression levels of isomiRs. The x-axis presents the total expression level. The ratio of the isomiRs was calculated using the number of the isomiRs in the bin divided the total number of isomiRs. For example, the ratio of the expression level isomiRs that lower than 1 is about 0.65. This implies that 65% of the isomiRs total expression level is lower than 1

A large amount of isomiRs were identified from the TCGA dataset. However, many isomiRs had to be removed since they were lowly expressed and had significant negative effects on the result. We defined in our dataset, that an isomiR was lowly expressed if the total expression level of the isomiR was relatively low in the dataset. The total expression level of isomiR was deemed the sum of the expression level of isomiR in all samples. In order to detect the distribution of total expression level of isomiRs, a histogram [29] of which the ‘bin’ of the bar graph equaled 1 was applied. Since the total expression level of isomiR was wide ranging, this histogram proved to be very large and therefore the complete histogram could not be displayed in this paper: the distribution of the total expression level less than 35 is shown in Fig. 2.

According to Fig. 2, about 65% of all isomiRs showed their total expression level was lower than 1. This implied that most of these isomiRs were lowly expressed. Further, it denoted that the distribution of the total expression level of isomiRs followed the exponential distribution. In order to remove these lowly expressed isomiRs, a null hypothesis method was applied. This null hypothesis states that: if the total expression level of an isomiR is very low, this isomiR is a noisy isomiR and should be removed. If the total expression level of an isomiR is very high, the null hypothesis can be rejected and this isomiR is not a noisy isomiR. For given *q* isomiRs and the expression level of each isomiR in all breast cancer patients, we first calculated the total expression level of each isomiR. The total expression level of *q* isomiRs are denoted as *TE*={*te*_1_, *te*_2_, …, *te*_*q*_}. The significance threshold *θ* of the competition score was calculated using the formula:

$$ \theta = \frac{q*\overline{TE}}{\chi^{2}_{1-\alpha/2}(q)} $$

Where $\overline {TE}$ is the mean of all the total expression level of isomiRs, $\chi ^{2}_{1-\alpha /2}(q)$ is the Chi-square with *q* defree of freedom, and *α* is the *p*-value. Here, the *p*-value was set at *P*=0.05. Only the isomiRs whose total expression level was smaller than this significance threshold *θ*, being viewed as lowly expressed, were removed.

### Calculating the weight of isomiR by improved mutual information

The mutual information is a powerful method in feature selection. Many mutual information-based feature selection methods have been developed and the performance has proven to be very good [21]. However, these methods has some limitations. Although some methods select a set of features that are very important for classification, they do not provide the weight of the feature. Some methods are applied from the data of which both the feature and the label are discrete or continuous data. However, these methods were not deemed suitable for this type of research. Therefore, an improved mutual information was developed to calculate the weight of each isomiR. This improved mutual information calculated the weight of each isomiR and measured the relationship between features and labels.

For any given expression profile of isomiRs, this expression profile has *m* isomiR *X*={*x*_1_, *x*_2_, …, *x*_*m*_}, *n* breast cancer patients *S*={*s*_1_, *s*_2_, …, *s*_*n*_}, and the subtype label of the patients *Y*={*y*_1_, *y*_2_, …, *y*_*n*_}. $x_{\tau }^{a}$ is defined as the expression level of isomiR *τ* in the breast cancer sample *a*. The min-max normalization method is applied to scale the expression levels of each isomiR between 0 and 1. The mutual information between an isomiR *x*_*τ*_ and breast cancer subtype *Y* is:

$$ I(x_{\tau},Y) = \frac{1}{n}\sum\limits_{i=1}^{n}\log\frac{f(x^{i}_{\tau}, y_{i})}{f\left(x^{i}_{\tau}\right)f(y_{i})} $$

Where $f\left (x^{i}_{\tau }\right)$, and *f*(*y*_*i*_) are the density function of isomiR and label, respectively. $f\left (x^{i}_{\tau }, y_{i}\right)$ is the joint density function of isomiR and label. Since the expression level of isomiR is continuous data while the label is discrete data, the density function of isomiR and label should be calculated by different equations:

$$ f\left(x^{i}_{\tau}\right)=\frac{1}{\sqrt{2\pi}n}\sum\limits_{j=1}^{n}exp\left(-\frac{\left(x^{i}_{\tau}-x^{j}_{\tau}\right)^{2}}{2}\right) $$

$$ f(y_{i})=\frac{1}{\sqrt{2\pi}n}\sum\limits_{j=1}^{n}exp\left(-\frac{d(y_{i}, y_{j})}{2}\right) $$

Where *d*(*y*_*i*_, *y*_*j*_) measures the distance between labels *y*_*i*_ and *y*_*j*_. If these two labels are continuous data, the distance between two labels can be calculated by Euclidean distance. However, the label in this research is discrete data. The distance of two labels cannot be calculated by Euclidean distance. *d*(*y*_*i*_, *y*_*j*_) is 0 if these two labels are the same, and it is 1 otherwise.

Since the improvement in calculating the distance between discrete labels, the mutual information is applicable for the dataset where the feature is continuous data and the label is discrete data. The joint density function $f(x^{i}_{\tau }, y_{i})$ can be calculated by using two-dimensional Gaussian kernel estimate:

$$ f(x^{i}_{\tau},y_{i}) = \frac{1}{2\pi n}\sum\limits_{k=1}^{n}exp\left(-\frac{D_{k}\left(x^{i}_{\tau},y_{i}\right)}{2}\right) $$

Where $D_{k}\left (x^{i}_{\tau },y_{i}\right) = \sqrt {\left (x^{i}_{\tau }-x^{k}_{\tau }\right)^{2}+d(y_{k},y_{i})}$.

This improved mutual information measured the relationship between features and labels. If the feature and the label have high co-relationship, the weight of the isomiR should be large. It implies that this isomiR is more important for the breast cancer subtype classification.

### Identification of isomiR biomarkers that classify breast cancer subtypes

A few key isomiRs, which have the highest weights, can distinguish between the different subtypes of breast cancer. These key isomiRs can then be used as breast cancer biomarkers, and they can be identified through these processes: sorting isomiRs by using their weights from large to small, then using the different top *N* isomiRs to evaluate the performance in the classification of breast cancer subtypes. The performance of this type of breast cancer classification will be raised with the increasing number of selected isomiRs. If the performance of classification by using top *N* isomiRs is not significantly raised compared to the performance by using top *N*+1 isomiRs, it implies that these *N* isomiRs are key isomiRs and can be viewed as biomarkers.

In this paper, the SVM [30] classifier was applied to classify different subtypes of breast cancer. According to Table 1, different subtypes of breast cancer have variable numbers of patients. Around 68% of breast cancer patients are ER *α*+HER2-, while nearly 4.4% of breast cancer patients are ER *α*-HER2+. This dataset is an imbalanced dataset and the SMOTE method was used to balance the data [31]. The receiver operation characteristic (ROC) curve is very popular to judge the discrimination ability of various statistical methods [32]. The area under ROC curve (AUC) measures the performance of the classifier [33]. Since this research is a multiclasses learning, macro-AUC of ROC was used to validate the performance of the classification [34]. Further, 5-fold cross-validation was applied to evaluate the results.

## Results and discussion

### Characterization of isomiRs identified in different subtypes of breast cancer

**Fig. 3:**
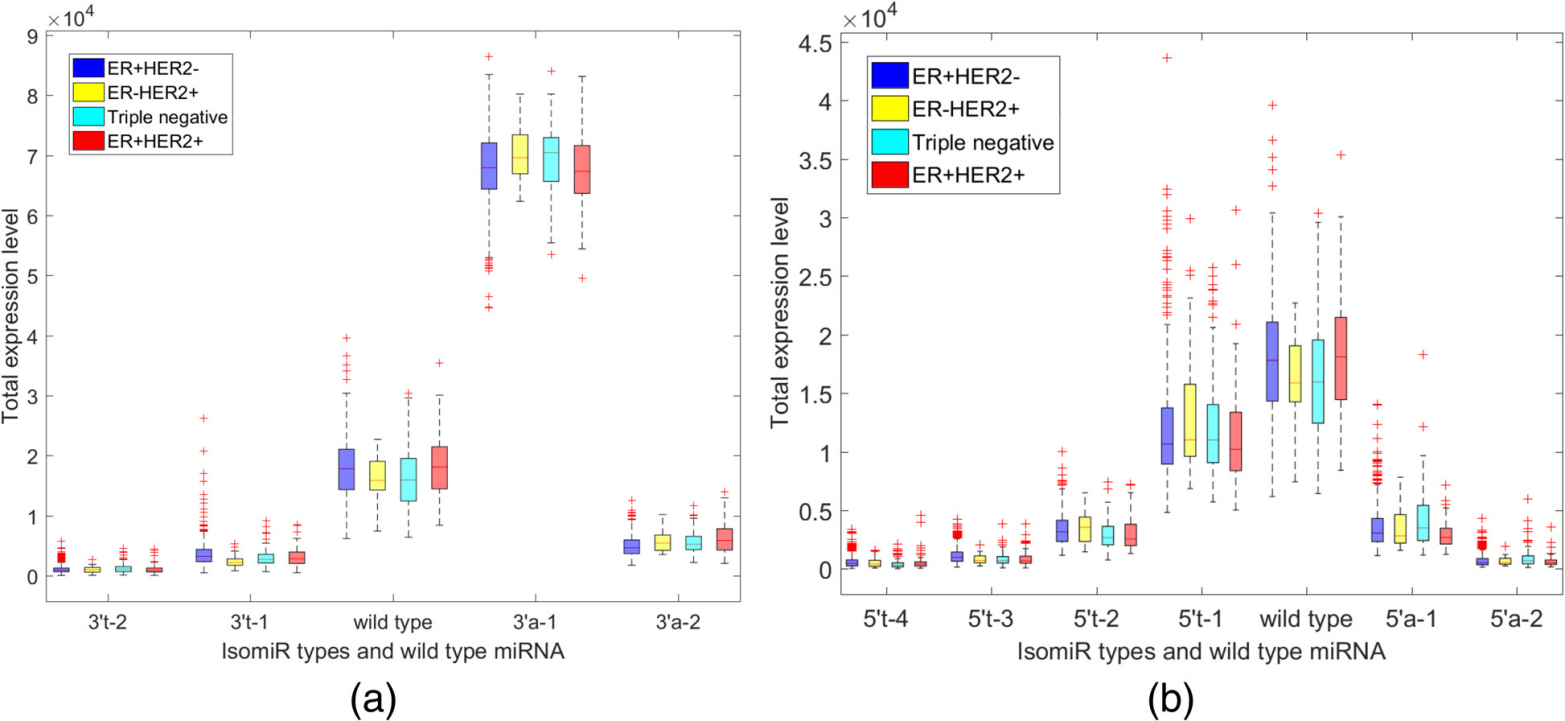
The distributions of isomiR modification types and their wild type miRNAs across different breast cancer subtypes. (**a**) 3^′^ isomiR and wild type miRNA distribution (**b**) 5^′^ isomiR and wild type miRNA distribution. Y-axis is the total expression level of isomiR (or wild type miRNA). The x-axis is the variant symbol. The variant symbol is divided into two parts by the sign (−). The left part of the sign (−) is the variate type at 3^′^ or 5^′^ position. The right part of sign (−) is the number of nucleotide added or trimmed at the 3^′^ or 5^′^ position

**Fig. 4:**
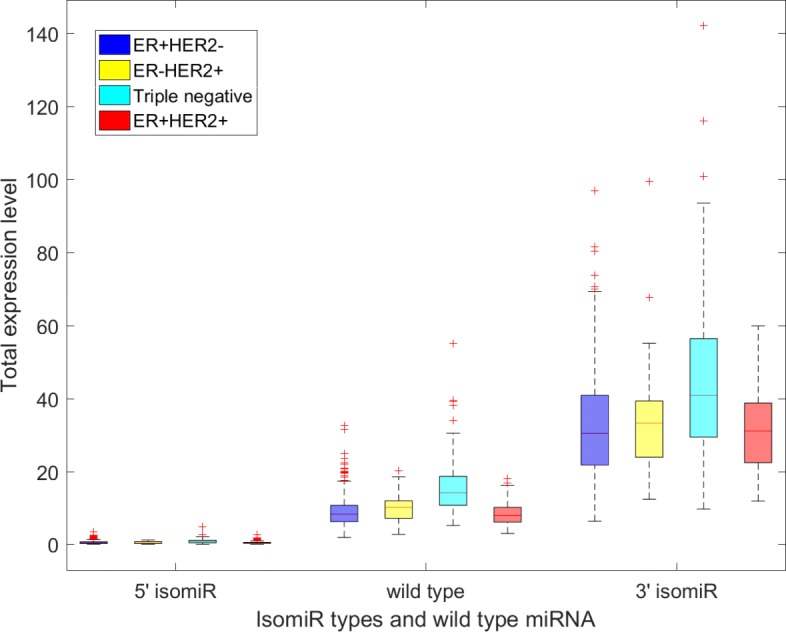
The distributions of miRNA has-let-7d-5p and its isomiRs across different breast cancer subtypes. The 3^′^ (5^′^) isomiR could have different lengths. The total expression level of 3^′^ (5^′^) isomiR is the sum of the expression level of different length of 3^′^ (5^′^) isomiR

In this study, 20134 different isomiRs were identified in the small RNA sequencing results of 698 breast cancer patients. However, most of the isomiRs were lowly expressed. Thus, we removed the lowly expressed isomiRs by using the null hypothesis method that was described in the subsection ‘Removal of lowly expressed isomiR’. Finally, 435 isomiRs, whose total expression level was larger than the significance threshold, were viewed as highly expressed isomiRs. Among these highly expressed isomiRs, 169 isomiRs were 5^′^ variant isomiRs and 266 isomiRs were 3^′^ variant isomiRs. These isomiRs are derived from 169 wild type miRNAs. The distribution expression of these isomiRs and their miRNAs across different breast cancer subtypes are shown in Fig. 3. In Fig. 3(a), only the total expression level of the isomiRs, of which one nucleotide is added at 3^′^ position, is larger than the expression level of wild type miRNA. While the expression level of the other 3^′^ isomiRs is lowly expressed compared with wild type miRNAs. In Fig. 3(b), the isomiRs, which trimmed one nucleotide at the 5^′^ position, has a similar expression level to the wild type miRNA. These two isomiRs (which added one nucleotide at 3^′^ position and trimmed one nucleotides at the 5^′^ position) may play vital roles in the breast cancer subtypes. Individual pre-miRNA may produce many different kinds of isomiRs and the expression level of isomiRs maybe higher than its wild type miRNA. Figure 4 displays the expression level of miRNA has-let-7d and its isomiRs across different breast cancer subtypes. We found that different kinds of isomiRs are produced during the miRNA maturation processes. Further, the expression level of isomiRs may be higher than the corresponding wild type miRNA.

### Identification of isomiRs that classify breast cancer subtypes

After the characterization of isomiRs in breast cancer, we calculated the weight of these isomiRs by using improved mutual information. Finally, we selected different numbers of isomiRs to compute their performance in the classification of breast cancer subtypes. The results and the Python source code of our algorithm can be downloaded from the website https://github.com/ChaowangLan/isomiRbreastsubtype.

**Fig. 5:**
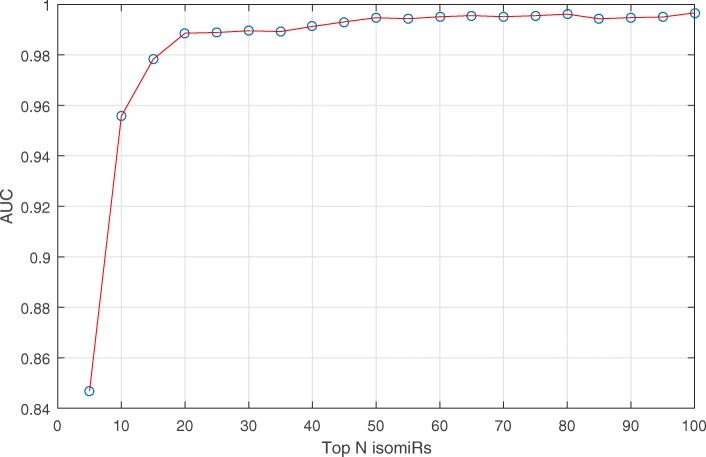
The performance of classification by using different number of isomiR. The x-axis is the number of isomiRs that are used to classify the breast cancer subtype. The Y-axis is the performance of the classification

According to Fig. 5, with the increasing number of isomiRs selected, the performance of the classification was improved. However, when the number of isomiRs was more than 20, the performance of the classification plateaued. Therefore, the number of key isomiRs for breast cancer subtype classification was 20. These 20 isomiRs are viewed as breast cancer subtype biomarkers. These isomiRs and their weights are listed in the second and third column of Table 2.

**Table 2:** The 20 isomiR biomarkers, their weights, and their ratios

Rank	IsomiR name	Weight	Ratios			
			ER *α*+HER2-	ER *α*-HER2+	Triple negative	ER *α*+HER2+
1	hsa-mir-106b-5p |5^′^a-1	1.86∗10^−3^	19.53	25.56	37.89	16.27
2	hsa-mir-28-3p |3^′^a-2	1.57∗10^−3^	0.51	0.65	0.87	0.53
3	hsa-mir-93-5p |3^′^a-1	1.46∗10^−3^	128.75	161.48	260.60	126.86
4	hsa-mir-106b-3p |5^′^t-1	1.45∗10^−3^	2598.00	3570.00	6163.00	2890.00
5	hsa-mir-106b-3p |3^′^a-1	1.37∗10^−3^	2702.00	3643.00	6395.00	2987.00
6	hsa-mir-17-3p |3^′^a-1	1.37∗10^−3^	1233.00	1601.00	3776.00	1251.00
7	hsa-mir-197-3p |3^′^a-1	1.19∗10^−3^	6.18	10.81	12.87	6.15
8	hsa-mir-92a-1-3p |5^′^t-1	1.14∗10^−3^	1.80	2.26	3.47	1.75
9	hsa-mir-146b-5p |3^′^a-1	1.13∗10^−3^	5.38	7.46	9.93	6.50
10	hsa-mir-210-3p |5^′^a-1	1.12∗10^−3^	15.69	40.67	37.39	20.70
11	hsa-mir-146b-5p |3^′^a-2	1.07∗10^−3^	11.12	15.06	19.60	14.63
12	hsa-let-7i-5p |3^′^a-1	1.03∗10^−3^	1.03	1.46	1.72	1.10
13	hsa-mir-210-3p |3^′^a-1	1.03∗10^−3^	206.94	513.97	497.48	272.43
14	hsa-mir-106b-5p |3^′^a-1	9.97∗10^−4^	46.36	56.38	85.98	39.33
15	hsa-mir-532-5p |3^′^a-1	9.60∗10^−4^	11.98	21.97	17.88	13.88
16	hsa-mir-93-5p |3^′^t-2	9.26∗10^−4^	6.85	7.89	13.59	6.31
17	hsa-let-7d-5p |3^′^a-1	8.80∗10^−4^	2.96	4.32	5.49	3.15
18	hsa-mir-27a-3p |5^′^t-1	8.51∗10^−4^	62.11	104.63	106.78	59.38
19	hsa-mir-22-3p |5^′^t-1	8.45∗10^−4^	0.04	0.05	0.05	0.04
20	hsa-mir-93-5p |5^′^t-1	8.45∗10^−4^	1.80	2.02	3.18	1.58

Among the isomiRs that faithfully characterize breast cancer subtypes, 7 isomiRs were identified as 5^′^ variant isomiRs and the other isomiRs were identified as 3^′^ variant isomiR. Most of these isomiRs were highly expressed compared to their corresponding wild type miRNAs. We calculated the ratio of the expression levels of these isomiRs and their corresponding wild type miRNAs in different subtypes of breast cancer. These ratios are listed in Table 2. If the expression level of an isomiR was larger than the expression level of its corresponding wild type miRNA, the ratio was larger than 1. Among these 20 isomiRs, only hsa-mir-28-3p | 3^′^a-2 and hsa-mir-22-3p | 5^′^t-1 were lowly expressed compared to their corresponding wild type miRNAs, the other isomiRs were more abundant. These results denote that many of these isomiR biomarkers are more highly expressed compared to their corresponding wild type miRNAs.

### Comparing the performance of improved mutual information to other feature selection methods

**Fig. 6:**
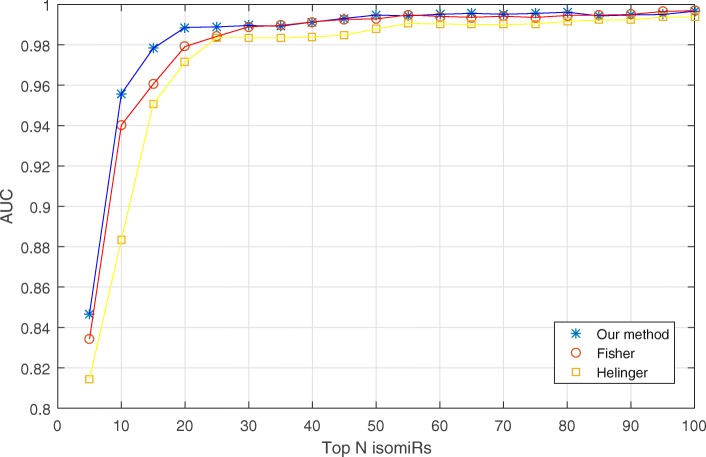
Comparison of our isomiR panel based novel method classification with other feature selection methods. The x-axis is the number of isomiRs that are used to classify the breast cancer subtype. Y-axis is the performance of the classification. The higher the AUC, the better the classification. Legend: the star represents the novel method described in this paper. The circle, and cross sign are the Filter method, and the Hellinger method, respectively

Many methods for feature selection have been developed. However, not all these methods are suitable for the dataset where feature is continuous data and label is discrete data. In this paper, we focused on comparing the performance of our novel method with two popular feature selection methods. One is the Fisher score and the other is the Hellinger distance-based method [26, 35]. The AUCs, calculated using the three different methods and using different numbers of selected isomiRs, are presented in Fig. 6. According to this figure, the AUCs show an increase with the raising of the number of selected isomiRs. However, if the number of selected isomiRs is larger than 30, the AUCs, which are calculated by these three methods, do not have significance changes. It indicates that the number of key isomiRs, by using these three methods, are lower or equal than 30. However, different methods identify different numbers of key isomiRs for breast cancer classification. The Fisher method identified 30 key isomiRs while the Hellinger method found 25 key isomiRs. Although fewer key isomiRs were discovered using Hellinger method, the AUC was found to be slightly lower than the Fisher method. Our method identified 20 key isomiRs that classify breast cancers, which is the lowest number of key isomiRs compared to the other methods mentioned, and the AUC was similar (nearly equal) to the Fisher method. It implied that our method can use fewer isomiRs as biomarkers to classify different subtypes of breast cancer.

### IsomiRs are superior biomarkers compared to protein coding gene expression-based approaches for the classification of different subtypes of breast cancer

Over the past decade, many studies have found that protein coding gene expression data can be used to classify breast cancer subtypes. For instance, Van and colleagues proposed that a 70-genes’ expression profile can use for identifying different subtypes of breast cancer [36], Parker and colleagues defined the PAM50 genes, which are the most famous biomarkers for breast cancer subtype classification [37], and Neve and colleagues also applied genes expression data for the classification of different subtypes of breast cancer [38]. Their research indicated that differentially expressed mRNAs can be used as breast cancer subtype biomarkers.

**Fig. 7:**
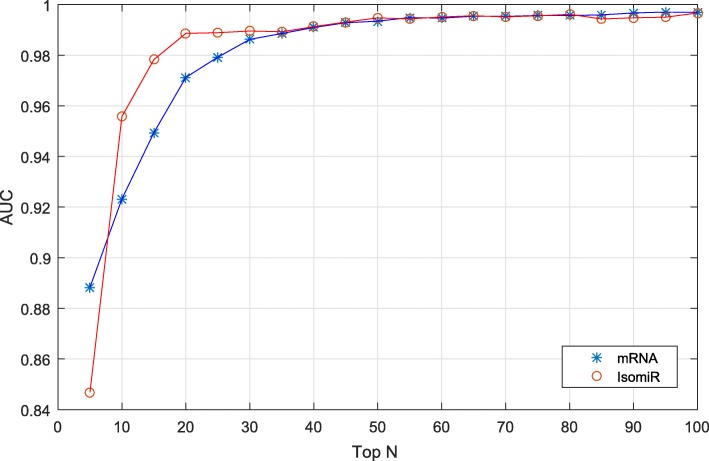
Comparison of isomiR and gene classification for breast cancer subtyping. The x-axis is the number of isomiR that are used to classify the breast cancer subtype. Y-axis is the performance of the classification. The higher the AUC, the better the classification. Legend: the star and circle present the classification using mRNA and isomiR, respectively

The TCGA database also provides the expression level of mRNAs in different subtype of breast cancer. Therefore, we can calculate if isomiR or gene expression-based profiling performs better for breast cancer subtype classification. Figure 7 presents the AUC by using isomiRs and mRNAs. According to the comparison in Fig. 7, the performance of breast cancer subtype classification using the expression of five mRNAs is very high (the AUC is near to 0.89). Direct comparison of isomiRs and mRNA (gene expression) clearly show that fewer isomiRs are needed to classify different subtypes of breast cancer compared to the number of mRNA (genes). With the increasing number of mRNA, the difference between the two classification methods is comparable, i.e. when the number of mRNA (gene classification) is more than 35, the AUC does not show any significant difference. Therefore, the number of key mRNA is 35, in comparison with isomiR, the key number is 20, showing fewer isomiRs can classify different subtypes of breast cancer. This experiment indicates that isomiRs also can be used as biomarkers for the classification of breast cancer subtypes and, importantly, fewer isomiRs can be used to classify different subtypes of breast cancer. These results strongly suggest that isomiRs are more suitable biomarkers compared to biomarkers based on protein coding gene expression profiles.

### IsomiRs may play important regulatory roles in different subtypes of breast cancer

Many studies have found that different categories of isomiRs have different functions in regulating biological processes. For example, 3^′^ isomiRs have low 3^′^ untranslated region stability and therefore, loose regulation of mRNAs [39]. 5^′^ isomiRs have slightly altered seed sequences compared to the corresponding wild type miRNAs; therefore, besides weakening the regulatory effect of the wild type miRNAs they can target mRNAs that are significantly different from the wild type miRNA targeted transcriptome [40]. Based on sequence similarities it is possible to predict potential mRNAs that are regulated by certain miRNAs [41, 42]and therefore, biological pathways that are influenced by miRNAs and their isomiRs. The elevated levels of isomiRs compared to their corresponding wild type miRNAs can also be used to predict changes in the regulation of gene expression in breast cancers that may well provide insight into the molecular mechanisms leading to breast cancer. We predicted that the presence of abundant 3^′^ isomiRs develop weakened regulatory effects on transcripts that are regulated by the corresponding wild type miRNAs. Thus, mRNAs that are regulated by the wild type miRNAs should show elevated expression levels when the expression level of isomiRs were significantly elevated. These targets that may be affected by the accumulation of 3^′^ isomiRs can be obtained from the miRWalker2.0 website (http://zmf.umm.uni-heidelberg.de/apps/zmf/mirwalk2/holistic.html). To predict potential targets for abundant 5^′^ isomiRs with modified seed sequence we used the miRDB website (http://www.mirdb.org/). In order to obtain the most likely targeted mRNAs, the score of the prediction target gene should be higher than 95 (the maximum score is 100).

After predicting the sets of potential mRNAs that are affected by the elevated miRNA isomiR levels, we wanted to characterize what molecular pathways may be changed in breast cancers. Enrichr (http://amp.pharm.mssm.edu/Enrichr/), which is a gene enrichment analysis web server, was applied to find out the Kyoto Encyclopedia of Genes and Genomes (KEGG) pathway of these target genes [43]. 104 KEGG pathways were identified as significant pathways (the *p*-value of these pathways were higher than 0.05) from this website. In this paper, we selected five pathways have been computed to be significantly affected by isomiRs to further discuss the function of the isomiRs in breast cancer. These 5 KEGG pathways are presented in Table 3.

**Table 3:** Five KEGG pathways which are relative to breast cancer progresses and subtype classification

KEGG name	*P*-value	Number of gene
Pathways in cancer	5.01∗10^−11^	96
p53 signalling pathway	1.29∗10^−6^	24
MAPK signalling pathway	1.20∗10^−5^	56
Insulin signalling pathway	3.16∗10^−3^	29
Estrogen signalling pathway	1.79∗10^−2^	20

The first two KEGG pathways in Table 3 are very important for analysis of breast cancer outcome [44]. This data suggests that isomiRs also play a vital role in breast cancer development. The clinical breast cancer classification is based on the hormone receptor status, some of these KEGG pathways are involved in regulating the hormone receptor status. For example, Neve’s research highlights that up-regulation of genes involved in insulin/MAPK signaling predicts response to Herceptin [38]. It implies that these two signaling pathways regulate the Herceptin status. According to the third and fourth line of Table 3, isomiRs were shown to influence 56 and 29 genes in MAPK and insulin signal pathways, respectively. Therefore, isomiRs could affect the Herceptin statue through these two pathways and lead to the development of different subtypes of breast cancer. We also identified the estrogen signalling pathway represented by 20 genes that is potentially affected by the isomiRs (Table 3). It implies that isomiRs could affect the expression of these genes to influence the estrogen receptor status. Above all, isomiRs may regulate the hormone receptor status via different KEGG pathways and therefore, affecting different breast cancer subtypes.

### Assessing the role of individual isomiRs in the regulation of breast cancer specific pathways

Next, we focused on the further analysis of six isomiRs that have potential to characterise/classify breast cancer subtypes. Dressman and colleagues pointed out that there are 18 genes that may delineate the role of estrogen receptor in breast cancer [45]. Transforming growth factor-beta type III receptor (TGFBR3) and serpin family A member 3 (SERPINA3) are two of these genes. Accordingly, the miRwalker 2.0 database, TGFBR3 is one of the potential target genes of hsa-let-7i-5p and SERPINA3 is the target gene of hsa-mir-197-3p. However, if one nucleotide is added to the 3^′^ position of these two miRNAs, then there is a possibility that these isomiRs cannot efficiently bind to the gene TGFBR3 and SERPINA3, respectively. This is because the longer sequence alters the stability of the miRNA and cannot inhibit the expression level of its target gene. Alternatively, 3^′^ isomiRs could be a sign of actively turned over miRNA that may have weakened regulatory functions. In the ER negative breast cancer tumors, most hsa-let-7i-5p wild-type miRNAs are altered to isomiRs hsa-let-7i-5p | 3^′^a-1 and hsa-mir-197-3p miRNAs are changed to isomiRs hsa-mir-197-3p | 3^′^a-1. Therefore, they are predicted to have a weakened affect to inhibit the expression level of TGFBR3 and SERPINA3 and these two genes are highly expressed in the ER negative breast cancer subtype. Similarly, these two genes are lowly expressed in the ER positive breast cancer subtype. Table 4 displays the average expression level of these two isomiRs in different subtypes of breast cancer. According to the average expression levels of isomiRs hsa-let-7i-5p | 3^′^a-1 and hsa-mir-197-3p | 3^′^a-1 in different subtypes of breast cancer, these two isomiRs are highly expressed in the ER negative tumors (ER *α*-Her2+ and triple negative breast cancer subtype) and lowly expressed in ER positive tumors (ER *α*+Her2- and ER *α*+HER2+ breast cancer subtypes).

**Table 4:** The average expression level of isomiRs and miRNA in each breast cancer subtype

IsomiR/miRNA name	Breast cancer subtype			
	ER *α*+HER2-	ER *α*-HER2+	Triple negative	ER *α*+HER2+
hsa-let-7i-5p |3^′^a-1	10.43	13.57	17.09	10.55
hsa-mir-197-3p |3^′^a-1	26.27	36.33	61.66	25.75

The 5^′^ variant isomiRs have distinct seed sequences compared to the corresponding wild type miRNAs; therefore, they may regulate a novel set of transcripts relative to the wild type miRNAs. Table 5 presents the predicted target genes of some 5^′^ variant isomiRs by miRDB database. The dysregulation of estrogen signalling pathway leads to ER positive breast cancer and therefore, the genes involved in this pathway may be the most attractive target for ER positive breast cancer treatment In the first line of Table 5, hsa-miR-93-5p | 5^′^t-1 may bind to gene SHC4. SHC4 is one of the gene involved in estrogen signaling pathway. The result implies that hsa-miR-93-5p | 5^′^t-1 may regulate SHC4 and dysregulate the estrogen signaling pathway. Furthermore, three 5^′^ variant isomiRs, which exhibited in the last three lines of Table 5, potentially bind to MAPK14, MAPK8, and RAP1B, respectively. These three genes are the part of the MAPK signaling pathway, which affects the Herceptin status. These results revealed that 5^′^ variant isomiRs may bind to genes that regulate hormone receptor status and therefore, lead to different breast cancer subtypes.

**Table 5:** 5 ^′^ variant isomiRs’ predicted target genes

isomiR	Predicted target mRNA	Score
hsa-miR-93-5p |5^′^t-1	SHC4	95
hsa-mir-27a-3p |5^′^t-1	MAPK14	97
hsa-miR-92a-1-3p |5^′^t-1	MAPK8	97
hsa-mir-106b-3p |5^′^t-1	RAP1B	95

## Conclusion

In this paper, we propose a novel method for identifying isomiR biomarkers for breast cancer subtyping from small RNA sequencing data. We first removed the lowly expressed isomiRs from the data sets. Then we calculated the weight of the isomiR by utilizing the improved mutual information. The improved mutual information measured the co-relationship between the expression level of isomiRs and breast cancer subtypes. The higher the co-relationship between isomiR’s expression and breast cancer subtypes, the more important the isomiR for breast cancer subtype classification. Further, this improved mutual information can be applied to the data set that the feature is continuous data and the label is discrete data. While the traditional mutual information cannot. Finally, the SVM classifier was applied to find specific isomiR biomarkers for classification of the different breast cancer subtypes. This method, proved to be more effective and efficient in identifying fewer key isomiRs needed for breast cancer subtyping in comparison to the Fisher and Hellinger methods. Importantly, in this study, we describe the enhanced identification of isomiR biomarkers for classification of breast cancer subtypes and, in addition, isomiRs were found to be superior biomarkers compared to classification based on mRNA gene expression for this type of classification. Further, applying this improved methodology, we identified individual isomiRs that may be key in the regulation of specific breast cancer pathways. There is great potential in exploiting these novel isomiR regulatory mechanisms as drug-targets for more personalized subtype breast cancer specific therapies.

Discovery of unique biomarkers in different breast cancer subtype is a challenge in research, especially since the regulation mechanism of different breast cancer subtypes is not yet fully understood. Our research provides a new way to explore the mechanism of breast cancer subtypes.
